# “Access to healthcare is a human right”: a constructivist study exploring the impact and potential of a hospital-community partnered COVID-19 community response team for Toronto homeless services and congregate living settings

**DOI:** 10.1186/s12913-023-10140-3

**Published:** 2024-04-25

**Authors:** Vivetha Thambinathan, Suvendrini Lena, Jordan Ramnarine, Helen Chuang, Luwam Ogbaselassie, Marc Dagher, Elaine Goulbourne, Sheila Wijayasinghe, Jessica Bawden, Logan Kennedy, Vanessa Wright

**Affiliations:** 1 Women’s College Hospital, 76 Grenville St., Toronto, ON M5S 1B2 Canada; 2 Neurology and Psychiatry, University of Toronto, 27 King’s College Cir, Toronto, ON M5S 1A1 Canada; 3 Gattuso Centre for Social Medicine, UHN, 200 Elizabeth St, Toronto, ON M5G 2C4 Canada; 4 Department of Family and Community Medicine, University of Toronto, 27 King’s College Cir, Toronto, ON M5S 1A1 Canada

**Keywords:** Community health, Hospital-community partnership, Homeless services, Integrated healthcare, Constructivist study

## Abstract

**Background:**

Individuals experiencing homelessness face unique physical and mental health challenges, increased morbidity, and premature mortality. COVID -19 creates a significant heightened risk for those living in congregate sheltering spaces. In March 2020, the COVID-19 Community Response Team formed at Women’s College Hospital, to support Toronto shelters and congregate living sites to manage and prevent outbreaks of SARS-CoV-2 using a collaborative model of onsite mobile testing and infection prevention. From this, the Women’s College COVID-19 vaccine program emerged, where 14 shelters were identified to co-design and support the administration of vaccine clinics within each shelter. This research seeks to evaluate the impact of this partnership model and its future potential in community-centered integrated care through three areas of inquiry: (1) vaccine program evaluation and lessons learned; (2) perceptions on hospital/community partnership; (3) opportunities to advance hospital-community partnerships.

**Methods:**

Constructivist grounded theory was used to explore perceptions and experiences of this partnership from the voices of shelter administrators. Semi-structured interviews were conducted with administrators from 10 shelters using maximum variation purposive sampling. A constructivist-interpretive paradigm was used to determine coding and formation of themes: initial, focused, and theoretical.

**Results:**

Data analysis revealed five main categories, 16 subcategories, and one core category. The core category “access to healthcare is a human right; understand our communities” emphasizes access to healthcare is a consistent barrier for the homeless population. The main categories revealed during a time of confusion, the hospital was seen as credible and trustworthy. However, the primary focus of many shelters lies in housing, and attention is often not placed on health resourcing, solidifying partnerships, accountability, and governance structures therein. Health advocacy, information sharing tables, formalized partnerships and educating health professionals were identified by shelter administrators as avenues to advance intersectoral relationship building.

**Conclusion:**

Hospital-community programs can alleviate some of the ongoing health concerns faced by shelters – during a time of COVID-19 or not. In preparation for future pandemics, access to care and cohesion within the health system requires the continuous engagement in relationship-building between hospitals and communities to support co-creation of innovative models of care, to promote health for all.

## Introduction

Individuals experiencing homelessness face unique physical and mental health challenges, increased morbidity and premature mortality [1, 2]. In Canada, it is estimated that 235,000 individuals experience homelessness annually, and 180,000 use emergency shelters each night [3]. Emergency shelters in Canada, also known as homeless shelters and congregate living facilities, support a diverse population of men, women, families, youth, newcomers, LGBTQ2S individuals and elderly [4]. Crowding within shared living spaces in shelters creates heightened risk for infectious disease outbreaks [5].

This heightened risk was demonstrated early in the COVID-19 pandemic when cases of COVID-19 surged among homeless people in Toronto. At one Toronto shelter, 40% of shelter residents tested positive for severe acute respiratory syndrome coronavirus 2 (SARS-CoV2) in a single onsite testing event in April 2020 [6]. People who are homeless are also at risk of more severe outcomes from COVID-19 infection. Individuals with a recent history of homelessness diagnosed with COVID-19, were 20 times more likely to be admitted to hospital, ten times more likely to require critical care and five times more likely to die of COVID-19, than those housed in Ontario communities [7]. Access to preventative population health measures demonstrate similar health disparity for the underhoused population. In September 2021, six months after COVID vaccines had become available to the Ontario population, 61.4% of individuals with a recent history of homelessness had received at least one COVID-19 vaccine, compared to 86.6% of the general population [8].

Primary care is the critical entry point for care related and unrelated to COVID-19 [9]. Access to primary care is essential to improving the health of those who are underhoused, yet a survey conducted in 2011, revealed less than half of Toronto’s homeless population identified having a primary care provider [10]. Models of primary care delivery for Toronto’s underhoused population include onsite clinics within homeless shelters, drop-in centres and mobile buses, all with varied funding models, catchments and ties to an array of organizational health partners, such as Inner City Health Associates, Parkdale Queen West Community Health Centre, and Safe Spaces & Care for the Homeless [11]. Despite the foundational role primary care plays within the health system, it was not identified as a formal platform for the provision of COVID-19 testing, infection control and COVID-19 vaccinations in the Toronto homeless population. Public Health Ontario privileged local public health authorities with vaccine delivery, rather than relying on existing primary care providers; this decision was controversial [12–14]. Rather, hospital, community health and social care organizations joined together in March 2020 and formed the Shelter and Congregate Support Coordination Table (SCSCT), created by Ontario Health, the regional health authority, in response to the surge of COVID-19 in homeless shelters.

Women’s College Hospital (WCH) is an ambulatory care facility situated in downtown Toronto. In March 2020, WCH set up one of Toronto’s 14 COVID-19 assessment centres (CACs) to facilitate and support free testing for SARS-CoV-2. The COVID-19 Community Response Team (CRT) was formed by a group of health care providers at WCH, in April 2020, as an extension of the CAC. The CRT routinely participated in SCSCT meetings and underwent cycles of adaptation to improve the model as the pandemic evolved [15]. The primary goal of the CRT was to support Toronto shelters, congregate living settings and supporting organizations across Toronto to manage and prevent outbreaks of SARS-CoV-2 using a comprehensive collaborative model through onsite mobile testing; supporting the management and prevention of outbreaks; and providing infection prevention and control training and guidance [15]. In total, CRT utilized this model of care through engaging with 49 shelter and congregate living sites from April 2020 to April 2021.

Over the course of the pandemic, SCSCT continued to provide operational guidance for health partners, particularly as COVID-19 vaccines became available. Geographic boundaries were eventually assigned for Toronto health partners to work within, based on their location. WCH was assigned to the mid-west part of Toronto. Fourteen shelter partners were identified for the WCH vaccine delivery program. Ten of these shelters had collaborative relationships with WCH prior to the pandemic.

Local vaccine procurement, administrative supports and processes were supported by a regional hospital partner, University Health Network. The WCH COVID-19 shelter vaccine program ran from March 2021 to September 2021 offering first, second and booster vaccine doses. Clinic teams ranged from utilizing eight clinicians to administer over 300 vaccines in a single clinic, to a solo clinician administering less than 20 vaccines, all within homeless shelters. Clinic frequency ranged from every one to three months. In total, 2300 vaccines were administered across the 14 sites. Clinic preparation was supported by the CRT shelter lead who liaised with a shelter administrator in advance of each clinic to set dates, review clinic flow, support any questions and, if possible, conduct a site visit in advance. Each supporting clinician (vaccinator) attended a single training on trauma informed care and cultural sensitivity before engaging in COVID-19 vaccine outreach. In addition, clinicians were briefed in advance of each clinic on shelter demographics, languages spoken, layout and relationship with WCH.

Studies examining hospital partnerships for community or population health have increased in the past five years [16]. Qualitative study findings in a systematic review on hospital-community partnerships for population health suggest these partnerships hold promise for breaking down silos, improving communication across sectors, and ensuring appropriate interventions for specific populations [16]. For example, implementation of a COVID-19 community-academic partnership model, in predominantly Black, Latinx, and otherwise racialized and/or low-income communities in San Francisco, California, was shown to be effective in creating a shared leadership and facilitating sustained linkages between partners [17]. Moreover, offering COVID-19 vaccines for the underhoused through known, hyperlocal and low barrier approaches, like community health workers and drop-in centres, has demonstrated increased trust among vaccine providers and recipients and vaccine uptake.

In this regard, the establishment of hospital-shelter partnerships offers a unique opportunity to provide culturally relevant, needs-based healthcare services to transitional housing settings. This research seeks to evaluate the impact and importance of the partnership model and its future potential. The overarching question addressed in this study is: What were the overall perceptions of shelter workers in this hospital-community partnership and strategy? Additional questions for analysis include: What were barriers, facilitators, and lessons learned throughout the process? And how can this partnership between the hospital and shelters be sustained in the future to fulfill needs beyond COVID-19?

## Methods

### Theoretical approach & study design

Epistemically, we approach this research with a health equity orientation, understanding that we have a responsibility as health professionals to provide expanded support to under-resourced individuals within the healthcare system. Through conversations with health partners, WCH acknowledged this need to deliver partnered programming (WCH shelter vaccine program) to combat the disproportionate effects of COVID-19 on homeless populations. Thus, this project is rooted from a social accountability standpoint; our research team believes hospitals should prioritize community partnerships to identify and deliver care based upon people’s needs within communities served.

This research project is located within a constructivist/interpretivist paradigm. In this paradigm, ontological assumptions are treated as ‘knowledge’ obtained by participating subjectively in a world of meanings created by individuals; all findings are seen as co-creations by both participant and researcher [18]. Constructivist grounded theory (CGT) is a qualitative research methodology used to understand and explore perceptions and construct theories about a social phenomenon, grounded in participants’ own experiences and words [19]. Theoretical data on the perceptions of hospital-community COVID-19 partnership are limited. In this study involving the voices of shelter administrators and staff, we chose CGT to explore and deepen analyses around perceptions and experiences of this hospital-community COVID-19 partnership strategy. CGT methodology is equipped to support a theory formation process most appropriately fitting the participants’ statements [20, 21]. CGT also considers and works to minimize the power asymmetries between researcher and participant, as well as showcase the knowledge asymmetries on both ends.

### Participant recruitment

This research was conducted with staff and administrators from shelters and congregate settings, whose organizations partnered with the WCH vaccine program to provide COVID vaccination for shelters between March 2021 to September 2021. As previously mentioned, prior on the onset of the WCH shelter vaccine program, COVID testing and infection, prevention and control education was also provided at many of these congregate living sites. Depending on the shelter organization, provision of COVID vaccines was either limited to clients only or directed at both clients and staff. To provide data richness and diversify participants’ experiences, we used maximum variation purposive sampling in our study [22]. The principal investigator (VW) reached out to shelter staff and administrators through email, inviting them to take part in a key informant interview with the research coordinator (VT). A total of 10 participants from 10 different organizations took part in this study. As a project involving quality improvement and program evaluation, this project was formally reviewed by institutional authorities at Women’s College Hospital (the Assessment Process for Quality Improvement Projects – APQIP) and has received Research Ethics Board approval.

### Data collection

Data collection occurred between April and August of 2022. After the initial participant recruitment email by the primary investigator, all further communication regarding the interview took place between the research coordinator (VT) and the participants. To prevent power differentials and seek raw answers about the WCH pandemic program, VT conducted all interviews, as they were not involved in design or delivery of the program. Participants who expressed interest were re-informed about the study by VT and emailed a consent form to read prior to the interview. A data collection form was also sent out to participants to complete, which captured demographic characteristics and WCH vaccine program-related data about the shelters (# of shelters with primary care partner on-site, number of residents, resident capacity before and after COVID (though it is still on-going), # of staff before and after COVID, referral mechanism to WCH vaccine program, whether vaccine education sessions were provided by WCH or other organizations, and vaccine uptake % as of October 2021).This was collected separately from the interview sessions to allow shelter administrators time to access their internal documents to gather data. Verbal consent was acquired at the beginning of the interview session. The 45-min sessions, conducted by VT, took place via Zoom© and were audio-recorded, transcribed, and de-identified using pseudonyms. To facilitate these sessions, VT used semi-structured interview guides developed by the research team. This guide centred around three key areas of inquiry: (1) Vaccine program evaluation and lessons learned (e.g., What were barriers, facilitators, and lessons throughout the process? How were shelter staff and clients impacted?); (2) Perceptions on hospital/community partnership (e.g., What were overall perceptions of this partnership and strategy?); (3) Opportunities forward (e.g., How can this partnership between hospitals and shelters be sustained in the future to fulfill needs beyond COVID-19. By the time of the last interview, theoretical saturation was reached, as no new conceptual information emerged.

### Data analysis

Aligned with the CGT data analysis method developed by Charmaz [23], this study approached data analysis through three stages: initial, focused, and theoretical. All data were imported into NVIVO 12 software for coding. VT and JR worked together to complete the first stage of analysis: initial coding. This is where line-by-line coding is performed, and information is gained inductively to create codes [23]. VT and JR individually worked through the 10 transcripts and tagged codes, which led to a creation of a joint codebook. VT and JR then came together to work on this codebook, discussing similarities and differences and potential conceptual groupings of the data. To clarify concepts and engage with the data in-depth, VT and JR made constant comparisons with questions such as “What is said, what do they mean, why is that said” [20]. This codebook was also shared with the research team (VT, JR, VW, SL) for feedback. After this, VT and JR coded three transcripts using the revised codebook to compare notes for meaningful coherence and interrater reliability. All transcripts were then coded using the codebook. Focused coding, the second phase of data analysis, consisted of VT and JR reviewing their codes and jointly identifying the emergence of analytically meaningful codes obtained from initial coding [19]. A meeting was held at this stage with the research team to debrief and discuss themes for further reflection and refinement. Lastly, VT, VW, and SL engaged in theoretical coding to further conceptualize the relationship between codes and partake in theory formation [23]. Memo notes were exchanged as a point of discussion about what stood out and what kept recurring to everyone as the core concept of the overall data analysis. This emerged as the core category “access to healthcare is a human right; understand our communities”. VT wrote the results, using assigned numbers for all participants.

### Reflexivity and quality criteria

To promote ongoing reflexivity throughout this research process and maintaining high quality rigour, the research team adopted the universal guidelines for reflexive thematic analysis [24]. This guideline outlines twenty critical questions to encourage deliberate reflection and engagement, specifically during data collection and analysis. The second (SL) and last authors (VW) were involved in the WCH pandemic program as a physician and nurse practitioner working at WCH. They have built relationships with the shelter organizations and have an innate attachment to this program as they were involved at the outset, with a passionate drive to deliver this community-based program during the COVID-19 crisis. Conscious of this, the research team had raw, reflexive conversations throughout this project to capture the impacts of the two authors’ experiences and how their positionality in this work may influence this study’s data analysis. Informed by CGT and our understanding of researchers’ roles within projects, we utilized the support of continuous research team debriefing, memo writing, and reflexive dialogue to reflect on how our codes are influenced by our team’s knowledge, beliefs, and experiences [19]. These practices enhanced credibility, consistency, and resonance of our study’s findings with respect to the participants’ experiences and overall context [21].

## Results & discussion

As described previously, a staff member from each shelter (*n* = 10) each filled out a data collection form, describing shelter demographic characteristics and the data related to the hospital vaccine program. Table 1 displays this compiled information, which is representative of data known at the time of interviews (April-August 2021).

**Table 1:** Shelter demographic characteristics and the hospital vaccine program-related data compiled from participants’ data collection form

	Special Characteristics of Facility	Primary care partner onsite?	Referral mechanism to hospital vaccine program	COVID Vaccine provision / education sessions provided by (other than the hospital partner):	Organizational Vaccine uptake % as of October 2021
P1	- Indigenous - Women - Refugee - Low barrier - Respite site	Y	- Hospital reached out - Pre-pandemic care partner - Self-referral	- Toronto Public Health - Quality Assurance / Practice Health Check - Hospital - Community Health Organization	98%
P2	- Indigenous - Women - Refugee - Low barrier - Shelter and drop-in program	N	Hospital reached out	- Toronto Public Health - Community Health Organization - Inner City Health Associates	76%
P3	- Refugee	N	Pre-pandemic care partner	None	85%
P4	- Women - Refugee - Low barrier - Undocumented	Y	Self-referral	None	100%
P5	- Refugee	N	Pre-pandemic care partner	- Toronto Public Health - Community Health Organization	85%
P6	- Refugee	Y	Pre-pandemic care partner - Hospital partner reached out - Self-referral	- Toronto Public Health - City of Toronto - Community Health Organization	79%
P7	- Refugee	N	- Hospital reached out	- Toronto Public Health - City of Toronto - Community Health Organization	100%
P8	- Refugee	N	- Hospital reached out	- Peer Navigators - Community Health Organization - Inner City Health Associates	100%
P9	- Women - Women who experience violence and abuse of any kind	Y	- Pre-pandemic care partner	None	85%
P10	- Refugee	Y	- Pre-pandemic care partner	- Peer Navigators	80%

Data analysis of the research findings revealed five main categories, 16 subcategories, and one core category. The core category is “access to healthcare is a human right; understand our communities”. The main categories are expanded COVID-19 response capacity, challenges identifying and managing outbreaks, barriers to the vaccine program, community-centred immediate shelter needs, and avenues for intersectoral relationship strengthening. Table 2 shows the categories, definitions, and subcategories of the hospital-community COVID-19 partnership model and strategy.

**Table 2:** Categories and definitions of the hospital-community COVID-19 partnership model and strategy

**Category** (focused coding)	**Definition**
**Expanded COVID-19 Response Capacity**	Assessing the hospital partner’s efficacy in supporting shelter to successfully respond to the COVID-19 pandemic.
**Challenges Identifying and Managing Outbreaks**	Shelter administrators’ concerns about navigating outbreaks through identification and management in in a congregate setting.
**Barriers to the Vaccine Program**	The ways in which the vaccine program was inaccessible for the shelter population and certain groups therein.
**Community-Centred** **Immediate Shelter Needs**	Current shelter/community-centred needs to be addressed.
**Avenues for Intersectoral Relationship Strengthening**	Long-term, systems-level opportunities where shelters, hospitals, and the general community can build stronger relationships.

### Core category: access to healthcare is a human right; understand our communities

The core category demonstrated the underlying perception that all shelter and congregate settings users experienced disproportionate effects of the COVID-19 pandemic. Shelter staff and administrators emphasized that access to healthcare is a consistent barrier for homeless populations, although it is a human right. The statement of the participant “Access to healthcare is a human right; understand our communities.” [P4] effectively captured the essence of all participants’ perspectives and therefore emerged as the core category of this study. Overall, participants explained that understanding the diverse communities and their needs is a requirement for care partners and for ensuring that hospitals work internally and externally to provide access to healthcare for all. With the pandemic, the barrier to accessing healthcare was exacerbated, but this hospital-based pandemic program helped alleviate some of its negative effects, as shown in the following categories below.

#### Main category: expanded COVID-19 response capacity

**Table:** 

**Subcategories** (initial coding)
• Increased access to resources
• Increased health knowledge (*IPAC* + *isolation policies*)
• A go-to trusted, credible partner

The Expanded COVID-19 Response Capacity category includes three subcategories: increased access to resources, increased health knowledge, and a go-to trusted, credible partner. In this category, participants assessed the hospital partner’s efficacy in supporting their shelter to successfully respond to the COVID-19 pandemic. Participants discussed the impact of the hospital partner’s support, in terms of what this hospital-community collaboration equipped them with, in order to respond to COVID-19. Ontario’s vaccination rollout, with an emphasis in Toronto, was slower than average, with confusing messaging and inconsistencies throughout various public health units [25, 26]. Under such provincial public health complexities, this local hospital-community partnership allowed for an increased access to resources, such as administering vaccines to clients within the shelter and receiving vaccines more quickly.

*Allowed us to offer our space for people to get and promote vaccines. Everyone (clients and staff) felt very confident and comfortable. There were doctors and nurses there to answer questions. It felt like they had all the support and information they needed. People would not have gone to get vaccines otherwise, unless we had physically accompanied each person [to an external location]. [P7]*

*We probably received vaccines quicker because of [the] partnership... A lot of the refugee homes aren’t city shelters; we’re independent NGOs that do this refugee home model; we’re not always on the city’s radar when it comes to public health initiatives. Ourhospital partner pushed to have vaccines delivered to refugee homes and it helped us getpeople vaccinated quicker. [P8]*

The hospital-community partnership model between shelters and WCH also increased shelter staffs’ health knowledge, with respect to IPAC (Infection Prevention and Control) support and isolation policies.

“*X sent photos of other clinic set-ups to show how they can be adaptable to any space.” [P7].*

*“Helped with IPAC support. Some staff brought up stuff during staff meetings. Anything I couldn’t answer, I asked *via* a quick email to our hospital partner, and they did their best to help with those pieces.” [P9].*

*“Increased our ability to be informed about COVID-19, so we can inform others. We reach out anytime we have questions about isolation policies and stuff, seek advice from health professionals, so this support is helpful in that sense.”* *[P6].*

During a time of confusion around infection prevention protocols and isolation practices, information originating from hospitals were seen as credible and trustworthy. Essentially, homeless shelters are considered housing facilities and resultingly, exist in a public health vacuum [4]. Despite not being included in IPAC standards, these shelters carry essential health functions that if not upheld would be detrimental to their inhabitants [4]. In many cases, shelters’ COVID-19 response capacity was strengthened by having a hospital partner as a first point of contact to answer shelters’ COVID-19-related information.

*“Good to receive COVID-19 information from a hospital. I feel it was a more trusted source, especially at the beginning of pandemic.” [P8]*

*“Made it a lot easier to run our shelter and get up to fuller capacity, less fear. Appreciative of our connection and their support.”* [P5].

*“Confidence in the vaccine because the hospital partner’s staff were well-educated and trustworthy people to the residents, so they were able to answer lots of questions. It promoted vaccine uptake for sure.”* [P7].

#### Main category: challenges identifying and managing outbreaks

**Table:** 

**Subcategories** (initial coding)
• Isolation challenges
• Movement of people in precarious circumstances

During the COVID-19 pandemic, shelter staff highlighted outbreak identification and management as a primary component of this hospital-community partnership strategy. Outbreaks were anticipated in this situation, yet it was unclear how shelters were to navigate through them, despite the hospital-community partnership strategy. Outbreak identification and management consist of two subcategories: isolation challenges and movement of people in precarious circumstances. Participants cited limited infrastructure and wait times for test results as contributing factors to isolation challenges.

*When we had the outbreak, it was really hard to contain it. It went on for a month –more and more people getting COVID. The set up that we have is such that it is hard to contain people for an extended period of time: for e.g., family of 4 sharing one hotel room. [P3].*

*The results at the hospital partner’s testing centre took very long. It was a challenge for a congregate setting, when we don’t know what to do when you don’t know results for a couple of days. We don't have isolation on site for clients that are symptomatic and waiting for results. We cannot refer families until we have a positive result. That's why so important to have test results very soon. [P10].*

Shelters have difficulty controlling the movement of people in precarious circumstances. This was crucial to limiting the spread of COVID-19 but proved impossible for families and people who must continue to go in-person to work and school for their livelihoods.

*One of the biggest challenges is, especially with pandemic, it’s very hard to control the movement of people (as a family shelter) - especially when school is open for example. Most of our clients are susceptible to getting infected with COVID-19 – managing this risk as a shelter is hard for us to control. We can’t control where and how people move around. [P3]*

#### Main category: barriers to the vaccine program

**Table:** 

**Subcategories** (initial coding)
• Inconsistent testing schedules
• Barriers to vaccine delivery
• Vaccine hesitancy
• Structural barriers for specific populations

The broad services offered by CRT over the course of the pandemic came with their own unique set of partnership challenges, particularly its shortcomings around vaccination and testing for the shelters’ clients. This category refers to the ways in which the program was inaccessible for the shelter population and certain groups therein. In addition to the frustrating testing wait times experienced by shelters, participants noted 4 main issues: inconsistent testing schedules offered onsite at the hospital, barriers to vaccine delivery, structural barriers for specific populations, and vaccine hesitancy. It should be highlighted here that these issues play out against a backdrop of ongoing systemic issues within the country’s healthcare environment. Though no individual hospital program may fix a structural issue, it is important to consider where special attention may be required when designing and carrying out such programs. For example, the hospital partner’s variability in testing schedules were disruptive for people in precarious circumstances and discouraged individuals’ participation.

*Often, testing hours change [at the hospital], and we wouldn’t know until after we sent someone…. difficult and confusing, would have been great to know in advance. There were challenging situations sometimes… [the hospital testing site] also might not have a very open/positive response to receive families who come near the end of the day.” [P10].*

With respect to vaccine delivery, many shelters had limited space and personnel and found it difficult to figure out the appropriate place to set up the vaccination site. When hospital partners arrived, participants said it created group gatherings, since many clients were curious with questions about COVID-19 and the vaccines. Shelter staff were needed to manage traffic flow in and out of the vaccination site, due to its inconvenient location in places like dining rooms. Some participants felt a pre-vaccine site visit would have helped alleviate this tension. All participants also felt this vaccine delivery process was unnecessarily administration-heavy on the shelters, who were expected to send paperwork with lists of client names for vaccination beforehand.

*I would say the capacity and manpower to organize the vaccine clinics is hard. The hospital partner has been great to come on very short notice, but it’s difficult at times. That’s what kept me back from hosting and organizing more clinics.” [P1]*

*The administration part, in terms of having the names and health card numbers, was heavy and challenging. Working with the vulnerable homeless population, it’s hard to pinpoint who was exactly going to be there in a drop-in situation on a given day. There was paperwork, locked key, and it had to be done X hours before and sent to X number of people. [P2].*

Contextualizing the shelter populations’ broader environmental concerns and recognizing the structural barriers individuals from specific populations face is critical to the overall success of hospital-community partnerships. Some unanticipated structural barriers for specific populations led to roadblocks and impeded shelter clients’ access to vaccination and care. Some affected groups were undocumented individuals, international students, out-of-province people – those without an Ontario Health Insurance Plan (OHIP) card.

*People without an OHIP card were afraid they’ll get sick with the vaccine, and then [they] don’t have support/immediate healthcare, or long-term supports… People who don't have OHIP, they don’t have access to emergency rooms right now because the Ministry of Health is not providing free services. [P4].*

Poor health care access is a long-standing fear for those who are undocumented or do not have OHIP as demonstrated in this comment above. It is important to recognize the depth of this commonly held belief in the context of COVID 19, where this understanding prevailed despite the Ontario government expanding health care entitlement to all people living in Ontario, Canada, with or without publicly funded health coverage on March 21, 2020, 10 days after COVID 19 was declared a global pandemic [27]. This perception speaks to the reality on the ground demonstrating practical access barriers; this may be an area where hospital partners could have made access issues clearer to their partners.

For clients with a history of trauma, participants hoped there could have been a more flexible approach to vaccination but understood the limitations within the current institutional structure that did not allow for this.

*Having flexibility in care would be ideal in helping/supporting certain clients with trauma. It would have been nice to make settings as non-clinical as possible would’ve been great because of people’s past negative experiences with the healthcare system. If we could offer vaccines at people’s bedspaces, that would’ve been great. [P1].*

Finally, vaccine hesitancy was the last subcategory of barriers to the vaccine program. Though the vaccine mandate motivated staff to get vaccinated, many clients were fearful and reluctant about getting the vaccine. Almost all participants agreed that the hospital partner’s vaccine education, specifically the 1-on-1 sit-downs with health professionals and webinars, supported increased vaccine uptake in their organizations. However, as of October 21^st^, 2022, 1.5 years after vaccine rollout, only 4/10 shelters reported an organizational vaccine uptake higher than 90% (Table 1). In shelters or congregate settings, movement cannot be strictly managed; thus there is a higher risk of infection. Therefore, vaccine uptake is a key preventative measure in managing the spread of COVID-19. Participants described the significance of understanding the historical discrimination clients faced by the healthcare system, and associated distrust in Western medicine and medical authorities.

*Like any other organization dealing with people from diverse groups, we are dealing with [a] lack of trust in vaccines, peoples’ experiences of medical discrimination, and so some clients are hesitant about getting vaccinated. It’s an ongoing, continuous discourse that all our partners must battle to minimize it. [P6].*

*We still need more education to combat vaccine hesitancy with our client groups, who are vulnerable and have complex medical issues. They’re afraid to take the vaccine… they’re transient, so they may say they’ll take the vaccine today, but they’re somewhere else tomorrow when the vaccines come. [P2].*

#### Main category: community-centred immediate shelter needs

**Table:** 

**Subcategories** (initial coding)
• Continuing pandemic support
• Mental health services
• Healthcare system navigation and access support

While speaking to participants about the efficacy of the vaccine program, several unaddressed, urgent shelter needs emerged. These community-centred exigencies were existing gaps that compounded shelters’ day-to-day pandemic requirements. Many shelters shared similar needs, which are categorized below into three main subcategories: continuing pandemic support, mental health services, and healthcare system navigation and access support. Continuing pandemic support, as requested by shelters, speaks to the unclear COVID-19 protocols since moving into fourth/fifth waves of the pandemic. Many participants spoke about looking for support in keeping up with changing protocols, at a time where pandemic fatigue is high and when there is still different information coming from different sources.

*Lots of questions and not enough clarity around COVID restrictions and what similar organizations should be doing (especially since we are not a shelter but still fall under congregate care). It was really easy before with simple provincial guidelines… now, grey areas around keeping people healthy and safe. [The hospital partner] could continue to offer consultations for those who are making those decisions… serve as consultants to review policies and make suggestions on policies and/or guidelines. [P7].*

*“Priority testing would be great for all shelters and congregate settings.” [P10]*

*A challenge is continuing to screen residents regularly. We don’t have enough staff to screen people. It got better in the middle, but now it sort of got degraded again with the fatigue. With fluctuating numbers and changing rules with COVID, it’s hard to know what we should be doing with COVID. [P8].*

The pandemic, through social isolation and widespread anxiety and depression, has detrimentally affected peoples’ mental health. Discussing the realities of how shelters serve many people with pre-existing mental health and addiction needs, participants called for mental health crisis support and intervention on-site to combat the increased number of crisis incidents during the pandemic. Many shelters specifically identified needing clinical intervention support, rather than counsellors.

*We need mental health supports – not just case managers or counsellors, more like clinical counselling and psychotherapy. A lot of the time, people are connected to counsellors when asked, but our staff are already counsellors. It’s the clinical piece that’s missing. Medical students will be coming on-site monthly to see residents and address any medical concerns they may have, but mental health supports are still needed. [P9].*

Many service care providers were forced to change their scope of functioning during the pandemic and shelters were no exception. However, this change wasn’t legitimized within the healthcare system. For instance, one participant recalled frustrating experiences during the pandemic, having to deal with individuals inappropriately discharged from hospitals to their shelter.

*“We are a shelter, but it also feels like a gateway to the shelter system at times, even though it’s not supposed to be anymore. COVID changes who we are and what we do; throughout the system, that’s it isn’t recognized exactly, so we get a huge number of inappropriate drop-offs and discharges from hospitals. We have clients sent in a taxi at midnight from the hospital wearing a hospital gown and no shoes, showing up clearly not well… And we end up sending them back by taxi. Anything that could help in terms of that process of medical discharges to the shelter system would be good; that’s a big system ask we have. [P2].*

Shelters also brought up the lack of information available around navigating the healthcare system and accessing supports, when it comes to both urgent and non-urgent care. This was underlined as key referral points hospitals, peer navigators, and healthcare professionals should share with local shelters in their geographic areas. The primary focus of many shelters lies in housing, and there is often little attention placed on health resourcing, solidifying partnerships, accountability and governance structures therein [4]. Recognizing this and working towards geographical alignment, with health partners, to meet these immediate shelter needs are essential to keeping communities’ needs at the centre of hospital-community partnerships.

#### Main category: avenues for intersectoral relationship strengthening

**Table:** 

**Subcategories** (initial coding)
• Health advocacy for individuals without OHIP
• Information-sharing tables
• Official partnerships
• Educate health professionals about systemic health inequities

Lastly, the final main category brings up long-term, systems-level avenues where shelters, hospitals, and the general community can build stronger intersectoral relationships. Hospital-community partnerships require sustainable change, commitment, and lasting support to strengthen their relationships to serve communities holistically. Four pivotal avenues were shared by participants: health advocacy for individuals without OHIP, information-sharing tables, official partnerships, and educating health professionals about systemic health inequities. Health advocacy for individuals without OHIP arose as a top ‘ask’ for many shelters. This could be related to the fact that the hospital partner had a specific clinical program that provides comprehensive medical services to newly arrived refugee clients. With COVID-19, there has been an increase in waiting times for attaining an appointment at this clinic and many refugee claimants not having a family doctor to depend on. Prioritization of this issue illustrates the value that health is a human right, not a status-based right.

*“Try to help us with encouragement and advocacy with different organizations, especially on behalf of people without OHIP.” [P4]*

*“Non-insured is always a challenge, and we have many women with no status in our shelter. We refer them to community health centres, but funding they have for this population always gets exhausted at the end of each year.” [P9].*

Almost all shelters outlined the potential of information-sharing tables as a tool for intersectoral relationship strengthening. Shelters wanted hospital engagement on a quarterly basis to check-in, ask about evolving community needs, and share relevant information that could support communities’ health needs, including safe injection sites, monkeypox anxieties, etc. With respect to information-sharing between hospitals and community partners, one participant eloquently detailed the need to not individually visualize each community shelter organization as a siloed entity.

*In addition to direct relationships, all of the refugee organizations have a really strong network and collaborate amongst us. When you think about collaboration between the hospital partner and our community, think more broadly than just individual organizations, think of us as a whole collective. A lot of the covid protocols and information that we got was collaborating amongst the various refugee houses. [P7].*

The need for formalized partnerships also came up as an avenue for stronger intersectoral relationships. This demonstrates a level of commitment that communities can expect from hospitals and provide a means for accountability. Some participants brainstormed the idea of designating an official liaison role between shelter and hospital systems, instead of participants having to haphazardly reach out to health professionals they knew from previous collaborative work.

Connecting directly back to the core category of understanding communities that hospitals serve, this final avenue consists of educating health professionals about systemic health inequities. Many participants are shocked by the lack of awareness health providers have about their clients’ realities. Without the knowledge of the landscape in which the healthcare system works and the inequities it perpetuates, participants explain that health professionals are not equipped to adequately support shelter clients.

*Helpful to have staff from the hospital partner who know this is a congregate setting and understand/be aware of challenges of the population they’re working with, to make sure they recognize the importance of situations & know limited access/availability to healthcare system for our clients. Perhaps then they could refer them accordingly or try ways to access other services even if not directly covered, or even collaborate to get more healthcare services. [P10].*

*It would be really amazing to have the support and all the hospitals to know what we are doing. We have had calls with social workers from hospitals who do not understand how we provide health care for people without OHIP. When doctors have the knowledge about lack of access to healthcare to people with OHIP, then doctors are more conscious/informed about how difficult it is for them. Knowledge is power. [P4].*

This study is unique in showcasing community perspectives in an official hospital-community partnership program during COVID-19. This hospital-based pandemic program demonstrates the importance of centring community voices in hospital-based community programs, as well as the breadth of knowledge gained by service providers when doing so.

During the COVID-19 pandemic, it was found that hospital service areas across the United States with a greater number of community partnerships (schools, community-based organizations, local agencies) had reduced case-fatality rates than those with fewer partnerships [28]. Our study similarly demonstrates increased access to resources and response capacity, as the organizational vaccine uptake across the 14 shelters in the study ranged from 76–100%. There is literature that has examined and identified successful factors for responding to COVID-19 in shelter-hospital partnerships, including an increase in resources, such as rapid access to testing, as well as support with restructuring physical spaces in line with IPAC and isolation policies (the latter being what we refer to as health knowledge in our study) [29]. However, our study explicitly lists the shelter workers’ perception and value of hospitals as a go-to trusted, credible health partner as crucial to this partnership and program, especially at a time of chaos and confusion during the pandemic. This building of trust and respect within an existing or new hospital-community partnership is important to a successful model.

As shown in a study in England investigating COVID-19 among people experiencing homelessness, outbreaks in homeless and congregate settings can lead to a high attack rate among the population, even when incidence remains low in the general population [30]. This means avoiding deaths is dependent on preventing transmission within such settings. Aligned with this, our study found that there were challenges identifying and managing outbreaks, despite the partnership in place. There was limited infrastructure and space for individuals to isolate while waiting on their test results. A review article on the prevention and mitigation strategies of respiratory infectious disease outbreaks among people experiencing homelessness suggests that interventions centered on reducing homelessness through income interventions, targeting macroeconomic factors, and the provision of adequate housing [31]. Hospital-community partnerships can minimize poor health outcomes and encourage bridging across sectors to promote health for all, but it remains clear that they are unable to eradicate broader system-level concerns without necessary structural change.

Building on the topic of existing system-level concerns, our study also revealed barriers to the vaccine program in ways in which the program was inaccessible for the shelter population and certain groups therein. Although our study’s barriers focused on the absence of an OHIP card for undocumented individuals and international students, and its impediment on their access to vaccination and care, other studies have shown that this is not an Ontario or even Canada-specific problem. A study in Rome detailed bureaucratic and organizational obstacles as similar impediments and showcased alternative approaches to cost-effective models that can reduce existing structural barriers to access diagnostic and preventive services for the homeless and undocumented population [32]. Thus, when designing and carrying out such hospital-community partnered programs, there must also be considerations of how to pay special attention to such gaps and simultaneously lobby for change in these areas.

Recently, a Toronto COVID-19 study examining the perspectives of people experiencing homelessness, healthcare workers, and shelter workers who cared for them, revealed how COVID-19 exacerbated the existing healthcare barriers for populations experiencing homelessness, including reduced shelter capacity, public closures, and lack of isolation options [33]. Our findings build on this by outlining current community-centred shelter needs and future avenues for intersectoral relationship strengthening. These suggestions should be taken into consideration when planning future hospital-community programs, with the recognition that the perspectives of shelter populations may differ depending on demographic context and location. Our findings highlight that the current Ontario healthcare system has many gaps and shortcomings when it comes to serving populations who are homeless. At a time of high anxiety and many health unknowns, hospitals were viewed as a trusted source for information, and this partnership model certainly provided benefits to siloed shelters without many institutional supports. Though structural change is necessary, hospital-based community programs in collaboration with shelters, can alleviate some of the ongoing health concerns faced by shelter populations – during a time of COVID-19 or not. For example, mental health crisis support and intervention on-site is a major community-centred immediate shelter need to combat the increased number of crisis incidents during the pandemic. A UK qualitative study exploring access to mental health and substance use support among individuals experiencing homelessness during COVID-19 noted that individuals experienced many forms of exclusion that were exacerbated during the pandemic, coupled with heightened mental health needs during this time of adversity [34].

In preparation for future pandemics and unanticipated health emergencies, access to care and cohesion within the health system requires the continuous engagement in relationship-building between hospitals and communities to support co-creation of innovative models of care, to promote health for all. The primary focus of many shelters lies in housing, and there is often little attention placed on health resourcing, solidifying partnerships, accountability and governance structures therein [35]. Recognizing this and working towards geographical alignment, with health partners, to meet these immediate shelter needs are essential to keeping communities’ needs at the centre of hospital-community partnerships. Hospital-community partnerships require sustainable change, commitment, and lasting support to strengthen their relationships to serve communities holistically.

No different from other research, this study has limitations. This research was conducted a year after the hospital-community partnered vaccine program took place, which was longer than an ideal follow-up time for interviews to take place. Participants had trouble remembering certain events; some individuals who had taken notes in a journal were able to better describe scenarios and share experiences than others. Additionally, despite the interviewer not having been involved with the vaccine program itself, their affiliation with WCH may have prevented participants to not be completed honest about the shortcomings of the program/partnership, especially since future collaborations were not yet formally established. With regards to the shelter demographic characteristics and the hospital vaccine program-related data compiled from participants’ data collection forms, it would have been interesting to learn more about how these characteristics impacted participants’ experiences and perceptions of the vaccine program. For instance, it would be helpful to know how a shelter’s referral mechanism to the hospital vaccine program may have impacted their overall perceptions and how this vaccine program may have impacted the shelter differently, depending on whether they had a primary care partner onsite. In our sample, 10/14 shelters had a relationship with WCH prior to the pandemic and this is important in contextualizing the presented information. Lastly, as a constructivist grounded theory study, there are limitations on the generalizability of knowledge constructed beyond this social context. These findings should be viewed as part of a larger puzzle and can be used to generate points of inquiry for further research in the field. Sampling from other geographical locations might further enhance our understanding of the phenomena explored in this study.

## Conclusions: policy and practice implications

In this study and through the process learnings of exploring the overall perceptions of shelter workers of a hospital-community partnership model, three key takeaways emerged for health(care) policy and practice:

1. ‘Health as a human right’ framework is an organizing principle in shelters but not necessarily in hospitals. How can hospitals adopt and integrate this framework at the policy level for internal and external operations/practice for a more equity-based approach to care?
2. For hospitals, there are gaps in knowledge about community and shelter realities. Ongoing formal partnering between hospitals and communities, through information-sharing tables and regular check-ins, is one way to bridge this gap.
3. Empowering shelter staff is crucial to the success of hospital-partnered programs and clinical interventions. This occurs through bidirectional knowledge transfer. Shelter workers regard hospital staff as knowledge holders. Hospital staff/clinicians are asked to recognize shelter workers as knowledge holders so to build a foundation for information exchange and co-design of interventions.

We hope this hospital-community partnership strategy adds perspective and inspires action in hospital administrators, healthcare professionals, and policymakers to move forward in a way that serves your communities’ population health needs. Finally, this project calls attention to the urgent context-specific exploration needed to advance official hospital-community partnerships, where there is an everlasting commitment and accountability.

## Data Availability

The results/data/figures in this manuscript have not been published elsewhere, nor are they under consideration by another publisher. Anonymized datasets used and/or analyzed during the current study may be made available from the corresponding author on reasonable request.
